# Cell-based receptor discovery identifies host factors specifically targeted by the SARS CoV-2 spike

**DOI:** 10.1038/s42003-022-03695-0

**Published:** 2022-08-05

**Authors:** Bushra Husain, Kobe Yuen, Dawei Sun, Shengya Cao, Jian Payandeh, Nadia Martinez-Martin

**Affiliations:** 1 Bayer AG, Portfolio and Assays group, Cologne, Germany; 2 Genentech, Oncology Biomarker development department, South San Francisco, CA USA; 3 Genentech, Structural Biology Department, South San Francisco, CA USA; 4 Genentech, Microchemistry, Proteomics and Lipidomics department. Receptor Discovery group, South San Francisco, CA USA; 5 Exelixis, South San Francisco, CA USA; 6 Regeneron Pharmaceuticals, Infectious Disease department, Tarrytown, NY USA

**Keywords:** High-throughput screening, Virology

## Abstract

Receptor-ligand interactions on the plasma membrane regulate cellular communication and play a key role in viral infection. Despite representing main targets for drug development, the characterization of these interactions remains challenging in part due to the dearth of optimal technologies. Here, we build a comprehensive library of human proteins engineered for controlled cell surface expression. Coupled to tetramer-based screening for increased binding avidity, we develop a high throughput cell-based platform that enables systematic interrogation of receptor-ligand interactomes. Using this technology, we characterize the cell surface proteins targeted by the receptor binding domain (RBD) of the SARS-CoV spike protein. Host factors that specifically bind to SARS CoV-2 but not SARS CoV RBD are identified, including proteins that are expressed in the nervous system or olfactory epithelium. Remarkably, our results show that Contactin-1, a previously unknown SARS CoV-2 spike-specific receptor that is upregulated in COVID-19 patients, significantly enhances ACE2-dependent pseudotyped virus infection. Starting from a versatile platform to characterize cell surface interactomes, this study uncovers host factors specifically targeted by SARS CoV-2, information that may help design improved therapeutic strategies against COVID-19.

## Abstract (web summary)

A high-throughput cell-based platform is developed for systematic investigation of receptor-ligand interactions and applied to identify cell-surface proteins that bind to SARS CoV-2.

## Introduction

Interactions between cell surface-expressed proteins on adjacent cells are key in sensing the extracellular microenvironment, initiating signaling cascades, and ultimately orchestrating intercellular communication. Their exposure on the cell surface and participation in key biological processes makes these in-trans receptor-ligand interactions a prime source of therapeutic targets. In fact, a large fraction of the drugs currently approved by the Food and Drug Administration (FDA) target membrane proteins, and future therapeutics aimed at blocking or modulating receptor-ligand interactions are likely to have important implications in preventing disease, including pathogen infection^1^. Despite representing an important source of potential therapeutic targets, the in-trans receptor-ligand networks that determine cellular communication, as well as pathogen infection have remained ill-characterized and underrepresented in current bases^2–4^. In particular, the receptors and cellular co-factors that mediate viral attachment and entry into the host cell are still unknown for many viral pathogens, despite these interactions representing ideal candidates for anti-viral development^5^. A more comprehensive evaluation of the host receptors that influence viral infection is essential to better understand their biological functions and clinical potential.

This discrepancy is in part explained by the biochemical intractability of the membrane-spanning proteins, and the difficulties to express and purify membrane proteins in their active conformation. In addition, the interactions between cell surface-expressed proteins are often characterized by low affinity, weak interactions, which challenge detection by commonly utilized methods^2,3^. Widely utilized technologies such as affinity purification-mass spectrometry (AP/MS) require stable interactions that can survive the stringent conditions for membrane extraction and washing and are optimally suited for the detection of abundantly expressed proteins, which is often not the case for membrane proteins. The more recent development of proximity labeling-based methods coupled with mass spectrometry has revolutionized our ability to detect transient protein interactomes with high sensitivity, however, these methods are generally restricted to *in-cis* interactions that take place in a cellular membrane^6^. On the other hand, protein microarrays and plate-based methods, which rely on the expression of membrane proteins as soluble ectodomains, are generally well-suited for the identification of in-trans interactions^7,8^. In combination with strategies that increase protein multimerization and binding avidity, these approaches have successfully identified membrane protein interactions in a number of biological systems^9–11^. Nevertheless, these methods are mainly focused on secreted or single transmembrane-containing proteins and can be relatively resource-intense in nature. In turn, the use of cDNA libraries for protein over-expression on cells overcomes some of these challenges by enabling receptor expression in the context of the plasma membrane, facilitating native conformation, post-translational modifications and receptor clustering that may be required for optimal protein functionality^4^. Notwithstanding their versatility and widespread use, variable or low protein expression at the plasma membrane as well as the scarcity of strategies to capture transient interactions in-trans still represent important challenges that have limited the applicability of the cell-based screening platforms.

Here, we implemented an automated cell-based platform for controlled protein expression on the cell surface, coupled to a tetramer-based screening method for enhanced detection of weak interactions in high throughput, which overcomes some of the limitations of current methodologies. To this end, we built an extensive library of human single transmembrane-containing proteins, engineered for controlled display on the plasma membrane. We demonstrate the applicability of this cell-based platform to characterize cell surface interactomes for diverse proteins, including the immune receptor B7-H3/CD276 or the secreted factor GDF15. Furthermore, we apply this technology to study the spike protein encoded by the severe acute respiratory syndrome coronavirus (SARS CoV-2), the causative agent of the coronavirus-induced disease 19 (COVID-19) and current global pandemic. Unbiased characterization of the single transmembrane protein interactome of the receptor binding domain (RBD) of the SARS CoV and SARS CoV-2 spike proteins led to the identification of the angiotensin-converting enzyme 2 (ACE2) as a common, high-affinity binder, as expected^12,13^. Interestingly, our results indicate that the previously identified SARS CoV-2 spike binder Neuropilin-2 (NRP2) also interacts with the SARS CoV RBD protein. By contrast, and notably, three additional host factors, Contactin-1 (CNTN1), IL1RAPL2 and IL12RB1 were identified as specific binding partners for SARS CoV-2 RBD, as well as the full spike ectodomain. Analysis of transcriptomics databases showed that these host proteins are expressed in a variety of human tissues where ACE2 levels are negligible. Moreover, we find that the novel SARS CoV-2 spike interactor CNTN1 is significantly upregulated in two large cohorts of COVID-19 patients. Surprisingly, using spike pseudotyped particles as a surrogate to study viral entry, we show that CNTN1 enhances ACE2-dependent infection. Together, these results suggest that CNTN1 is a previously unappreciated host co-receptor for SARS CoV-2 that may potentiate viral infection.

In sum, here we present the optimization of a versatile cell-based platform for optimal detection of in-trans protein-protein interactions with enhanced sensitivity and throughput. The technological advance presented in this report offers an important tool to enable the systematic evaluation of receptor-ligand networks for architecturally diverse proteins, including immune receptors, secreted proteins and virus-host interactions. The identification of host factors that are specifically targeted by the spike protein provides valuable insights into basic aspects of SARS CoV-2 infection that may ultimately inform the development of anti-viral therapeutics and vaccination strategies.

## Results

### Optimization of a cell surface-display platform for enhanced receptor discovery

Protein interactions in the extracellular milieu are often characterized by transient, low-affinity binding (K_D_ in the micromolar to the millimolar range) that challenge identification by the most commonly used approaches. To facilitate detection of such interactions, several methods have been developed that rely on fusion domains or tags that promote ectodomain multimerization, increasing binding avidity and thus enabling the detection of weak receptor-ligand binding events. Here, we took advantage of the interaction between biotin, a small and stable label, and streptavidin, which binds up to four biotin molecules with extraordinarily high affinity. Based on this widely utilized pair, we sought to implement a simple and minimally disruptive approach to enable ectodomain multimerization while also allowing large-scale screening and sensitive detection of binding events using a fluorescent readout. As proof-of-concept, we first focused on the immune receptor PD-L1/CD274 and the poliovirus receptor PVR, which are known to recognize multiple ligands with binding affinities that range from nanomolar to micromolar^14,15^. PD-L1 and PVR ectodomains were expressed as recombinant avi-tagged proteins and subsequently biotinylated in vitro using the BirA enzyme to ensure site-directed biotinylation and thus avoid any further protein modification that could interfere with ligand recognition. Next, the biotinylated ectodomains where tetramerized using fluorescently-labeled streptavidin to facilitate the detection and quantification of receptor-ligand interactions.

**Fig. 1:**
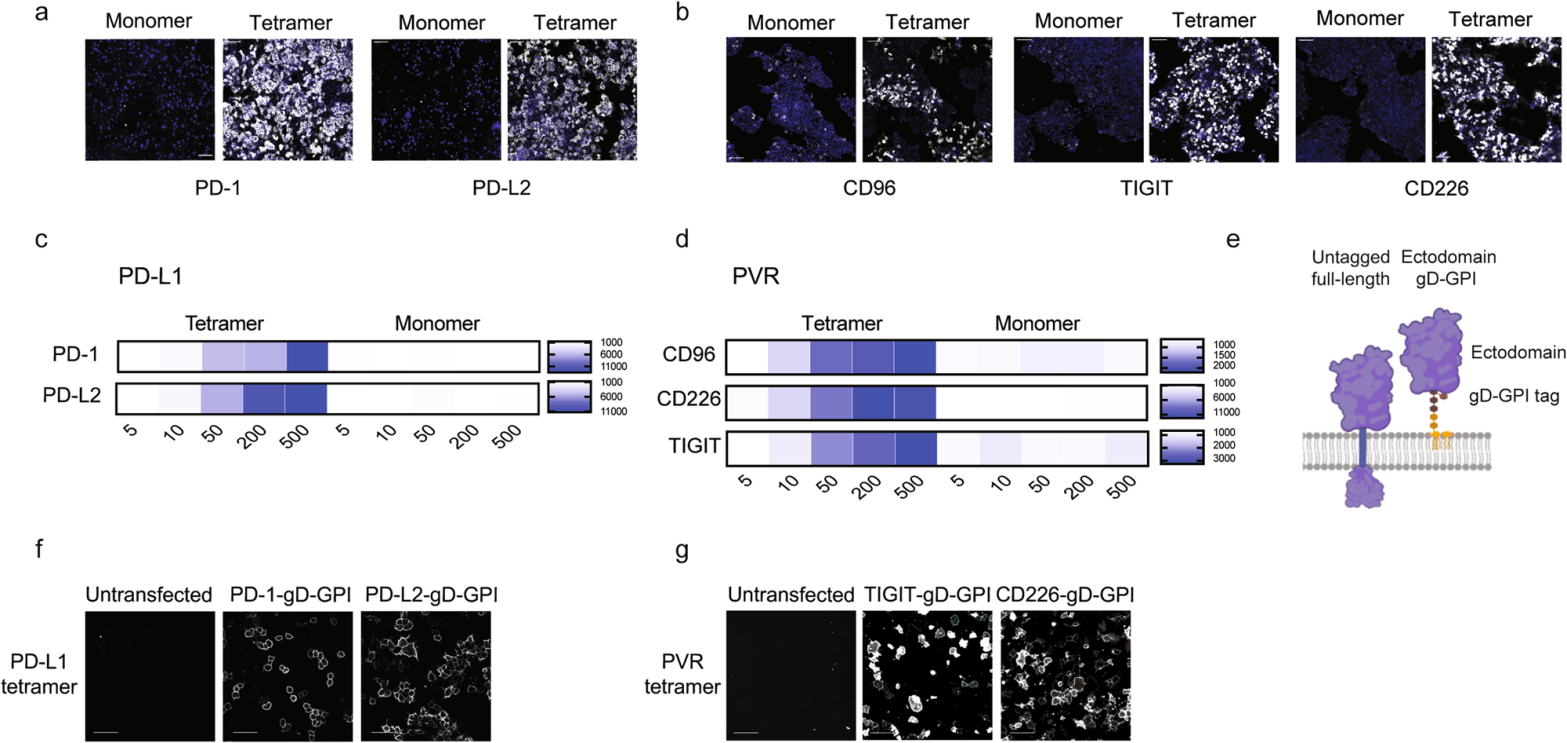
Optimization of a cell-based platform for enhanced receptor-ligand discovery. Representative images of (**a**) PD-L1 or (**b**) PVR ectodomain binding to cells expressing the indicated binding partners as full-length, untagged receptors. Tetramer binding to the cell surface is represented in white, nuclei are depicted in blue. Ectodomain binding at 100 nM is shown in the representative images. Scale bar = 100 μm. Quantification of (**c**) PD-L1 or (**d**) PVR ectodomain binding to cells expressing the indicated binding partners. PD-L1 or PVR were tested as recombinant monomeric or tetrameric ectodomains at the indicated concentrations (nanomolar) and binding to the cell surface was represented as normalized fluorescence intensity. **a** Schematic representing the tagging strategy used to generate the ectodomain-gD-GPI library. Binding of (**f**) PD-L1 or (**g**) PVR ectodomain tetramer to the indicated binding partners on the cell surface, expressed as ectodomain-gD-GPI proteins. Scale bar = 50 μm.

First, to assess the effect of ectodomain tetramerization on the detection of binding partners, recombinant monomeric or tetrameric PD-L1 or PVR ectodomains were tested for binding to cells expressing specific binding partners. We tested PD-L1 ectodomain binding to the surface of cells expressing PD-1 or PD-L2 proteins (Fig. 1a), whereas PVR was assayed for binding to the cell surface of CD96-, TIGIT- or CD226-expressing cells (Fig. 1b). The recombinant ectodomains were incubated with the cells on ice to avoid potential internalization processes and subsequently washed with buffer to minimize any assay background. Tetramerization of the query protein ectodomain enhanced detection of the relevant interactions relative to the monomeric ectodomain assayed at the same molar concentrations, including micromolar affinity interactions such as PD1-PD-L1 (Fig. 1a). Analysis of the fluorescent signal also enabled relative quantification of ectodomain binding to the cell surface (Fig. 1c, d).

The use of cDNA encoding full-length receptors that are transiently transfected in cells of interest generally enables protein expression on the cell surface and facilitates the study of the interactions in the context of the plasma membrane. However, poor or uncontrolled receptor expression at the cell surface represents a main challenge that can lead to high false negative detection rates and underrepresentation of transient interactions. To improve these aspects while enabling a systematic assessment of receptor-ligand interactions on the plasma membrane, we generated a collection of membrane proteins, expressed as ectodomains fused to a glycoprotein D (gD) tag and a glycosylphosphatidyl-inositol (GPI)- linker (referred to as ‘ectodomain-gD-GPI’). This receptor tagging strategy facilitates protein targeting to the plasma membrane, via the GPI link, as well as quantification of protein expression on the cell surface, measured by gD tag staining (Fig. 1e). We first tested binding of PD-L1 ectodomain, tetramerized using fluorescently-labeled streptavidin, to cells expressing PD-1 or PD-L2, expressed as ectodomain-gD-GPI constructs that were transiently transfected in cells. These interactions were readily detected on the cell surface, including sensitive detection of the relatively weak interaction with PD-1 (Fig. 1f). Similarly, the PVR tetramer bound to the surface of cells expressing the known interactors TIGIT or CD226, expressed as ectodomain-gD-GPI proteins (Fig. 1g).

**Fig. 2:**
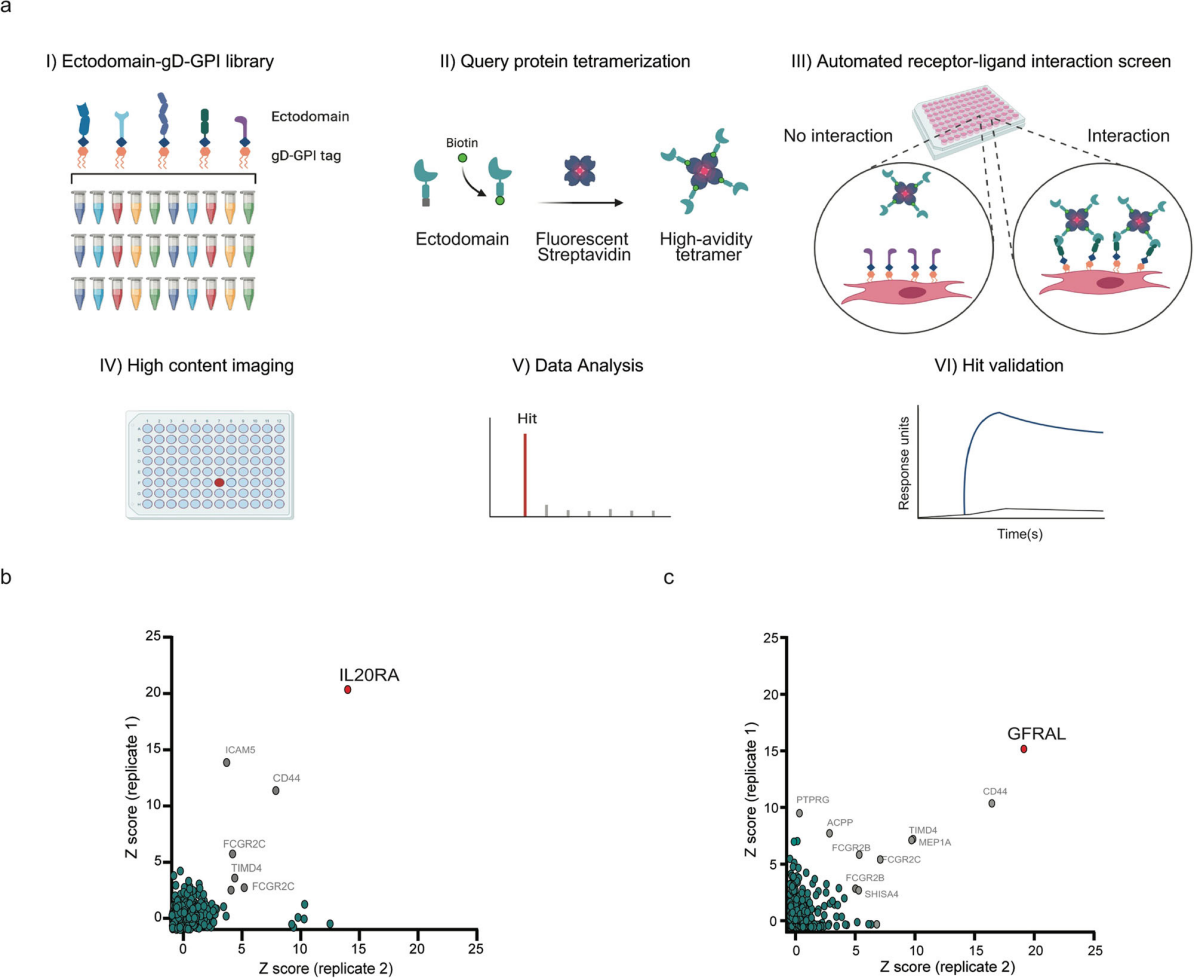
An automated cell-based platform for enhanced receptor-ligand discovery. **a** Schematic representation of the automated cell-based platform for receptor-ligand discovery. (I) A library consisting of ≈1,200 unique single transmembrane-containing receptors and selected isoforms, expressed as ectodomains fused to a gD-GPI tag, was generated. (II) The query protein, expressed as avi-tagged ectodomain was biotinylated and subsequently tetramerized using fluorescent streptavidin for increased binding avidity. A workflow for automated transfection, cell surface binding analysis, and high content imaging was implemented. (III) Individual receptors were transfected in mammalian cells, and binding of the query protein tetramer to the cell surface was detected by measuring fluorescent signal. (IV) Images from individual wells are acquired using a high-content microscope. (V) Fluorescent signal intensity is calculated for each image and represents query protein binding to each receptor expressed on the plasma membrane. (VI) Following data analysis and hit calling, new interactors are confirmed using orthogonal techniques such as surface plasmon resonance. Unbiased screening using the receptor-ligand discovery platform identifies expected binding partners for the (**b**) receptor B7-H3/CD276 and (**c**) the secreted factor GDF15. Intersection plot represents binding of each receptor in the library, in duplicates. Each circle represents query protein binding to a unique receptor in the library. Unique high-scoring hits are shown in red circles. Hits shown in grey circles are empirically determined non-specific binders.

Having shown that the ectodomain-gD-GPI proteins are amenable for detection of receptor-ligand interactions on the cell surface, we decided to generate a large library encompassing most single transmembrane-containing proteins in the human genome, expressed using the same tagging strategy. Taking advantage of this ectodomain-gD-GPI library and the tetramer-based screening strategy, we then sought to develop a platform for high throughput receptor discovery. To this end, we implemented a small-scale, automated cell transfection and semi-automated screening format for larger throughput and increased reproducibility, coupled to high content imaging to quantify staining on the cell surface (Methods and Fig. 2a). First, we assessed the overall expression of the library of ectodomain-gD-GPI proteins taking advantage of the gD tag for facile detection of protein expression on the cell surface. Notably, from over 1200 unique single transmembrane-containing proteins analyzed for cell surface expression, medium to high cell surface expression levels were achieved for over 75% of the single transmembrane-containing proteins, whereas less than 10% of the proteins did not show the detectable expression on the plasma membrane under the experimental conditions utilized (Supplementary Fig. 1). These results suggest that most of the receptors in the library are displayed on the cell surface and available for interaction with the potential in-trans binding partners.

Next, to determine the sensitivity and applicability of this platform to study diverse extracellular protein interactions, we first tested B7-H3, an immune receptor deorphanized only recently^11^. As described, B7-H3 was expressed as a recombinant avi-tagged ectodomain for site-specific biotinylation, followed by tetramerization using fluorescently-labeled streptavidin. The B7-H3 ectodomain tetramer was then screened against the gD-GPI-tagged library using the automated cell-based platform (Fig. 2a). Notably, when B7-H3 was screened for cell surface interactors in an unbiased fashion the interleukin-20 receptor subunit alpha (IL20-RA) was detected as the only specific high scoring hit, in agreement with recent findings^11^. (Fig. 2b). Next, to further probe this platform, we screened the unrelated protein GDF15, a secreted factor that belongs to the transforming growth factor-beta superfamily. In this case, GDF15 was expressed as a full-length protein fused to an avi-tag, followed by biotinylation and tetramerization. These efforts identified the receptor GFRAL as the only specific and high scoring hit for GDF15 (Fig. 2c), as previously observed^16^ and confirming the applicability of the new platform to study diverse query proteins.

### The SARS CoV-2 RBD single-transmembrane interactome

**Fig. 3:**
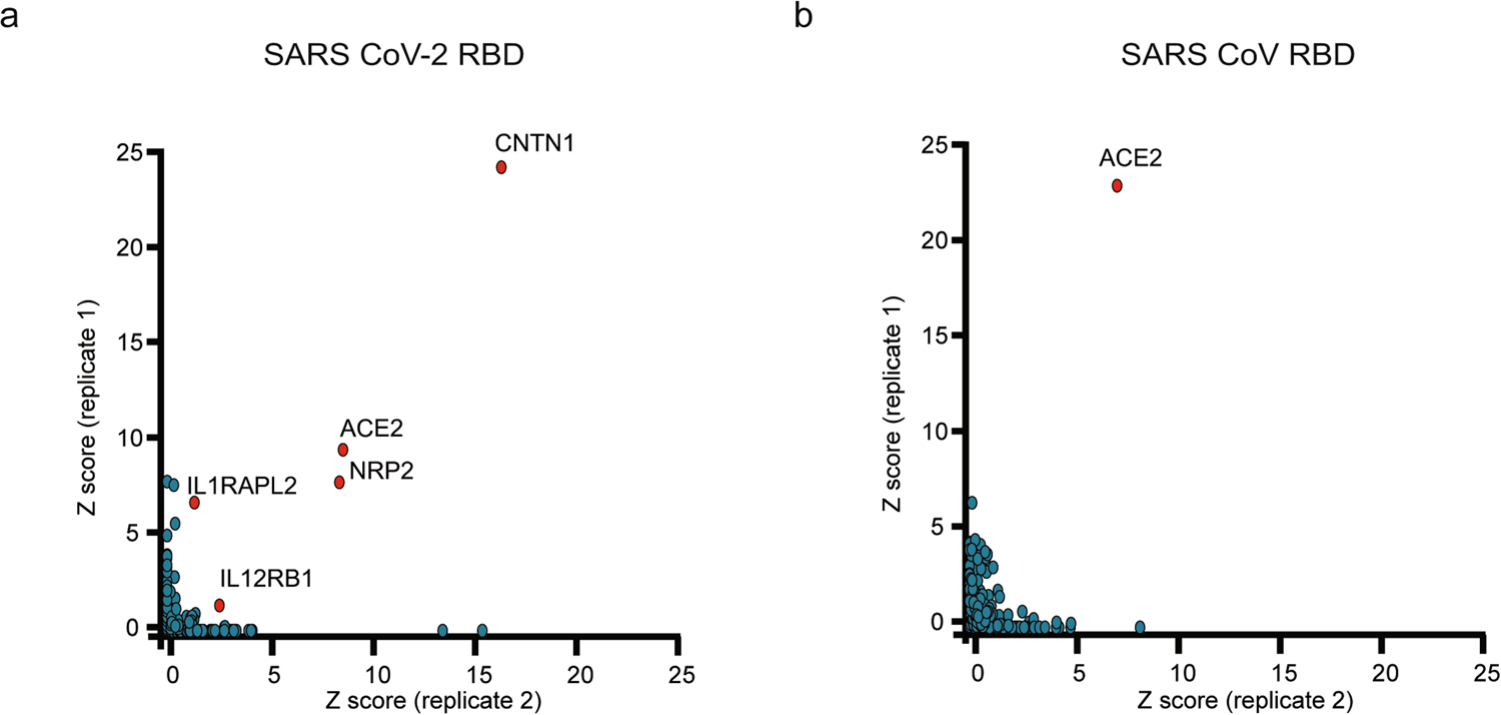
Single transmembrane protein interactome screening identifies host cell surface factors specifically targeted by the SARS CoV-2 RBD. **a** Intersection plot representing binding of the RBD of the SARS CoV-2 spike protein to the library of single transmembrane-containing human proteins, screened in duplicate. ACE2, IL12RB1, IL1RAPL2, CNTN1 and NRP2 were identified as specific, high scoring hits. **b** Intersection plot representing binding of the RBD of the SARS CoV protein to the library of single transmembrane-containing human proteins, screened in duplicates. ACE2 was identified as high scoring hit.

Multitude of studies have shown that similar to the related SARS CoV, SARS CoV-2 utilizes the host protein ACE2 as a main receptor for viral entry, through an interaction mediated by the receptor binding domain (RBD) of the spike protein. Subsequent membrane fusion is facilitated through spike protein priming by host cell proteases, including TMPRSS2 and serine proteases^13^. Although COVID-19 patients primarily manifest symptoms in the respiratory tract, ACE2 levels are low throughout the respiratory system relative to other tissues, suggesting the existence of additional host factors that may facilitate viral entry and/or attachment upon direct interaction with the spike protein. Thus, we sought to utilize our platform to evaluate the single transmembrane protein interactome of the SARS CoV-2 spike, focusing on the RBD that is responsible for the interaction with ACE2. Interestingly, RBD protein screening identified ACE2 as a prominent hit, as expected, alongside Neuropilin-2 (NRP2), recently described as a SARS CoV-2 spike binding partner (Fig. 3a)^17^. Surprisingly, three additional cellular receptors were identified as high-scoring hits for SARS CoV-2 RBD, including the interleukin-12 receptor subunit beta-1, IL12RB1, the interleukin-1 receptor accessory protein-like 1, IL1RAPL2, as well as the nervous cell surface adhesive protein Contactin-1, CNTN1. Next, to assess the host factor targeting specificity for SARS CoV-2 and carry out an unbiased evaluation of the highly related SARS CoV spike protein, similar screens were performed to characterize the single transmembrane receptor interactome of the RBD of the SARS CoV spike. Notably, these assays identified ACE2 as the only top-scoring hit (Fig. 3b). The cell surface proteins CNTN1, IL12RB1 or IL1RAPL2, identified as putative binding partners for SARS CoV-2 RBD, were not detected in these screens. Although weak binding to NRP2 was observed for the SARS CoV RBD upon close inspection of the images, this putative interactor was not detected as high scoring hit due to the relatively weak RBD binding to the NRP2-expressing cells.

**Fig. 4:**
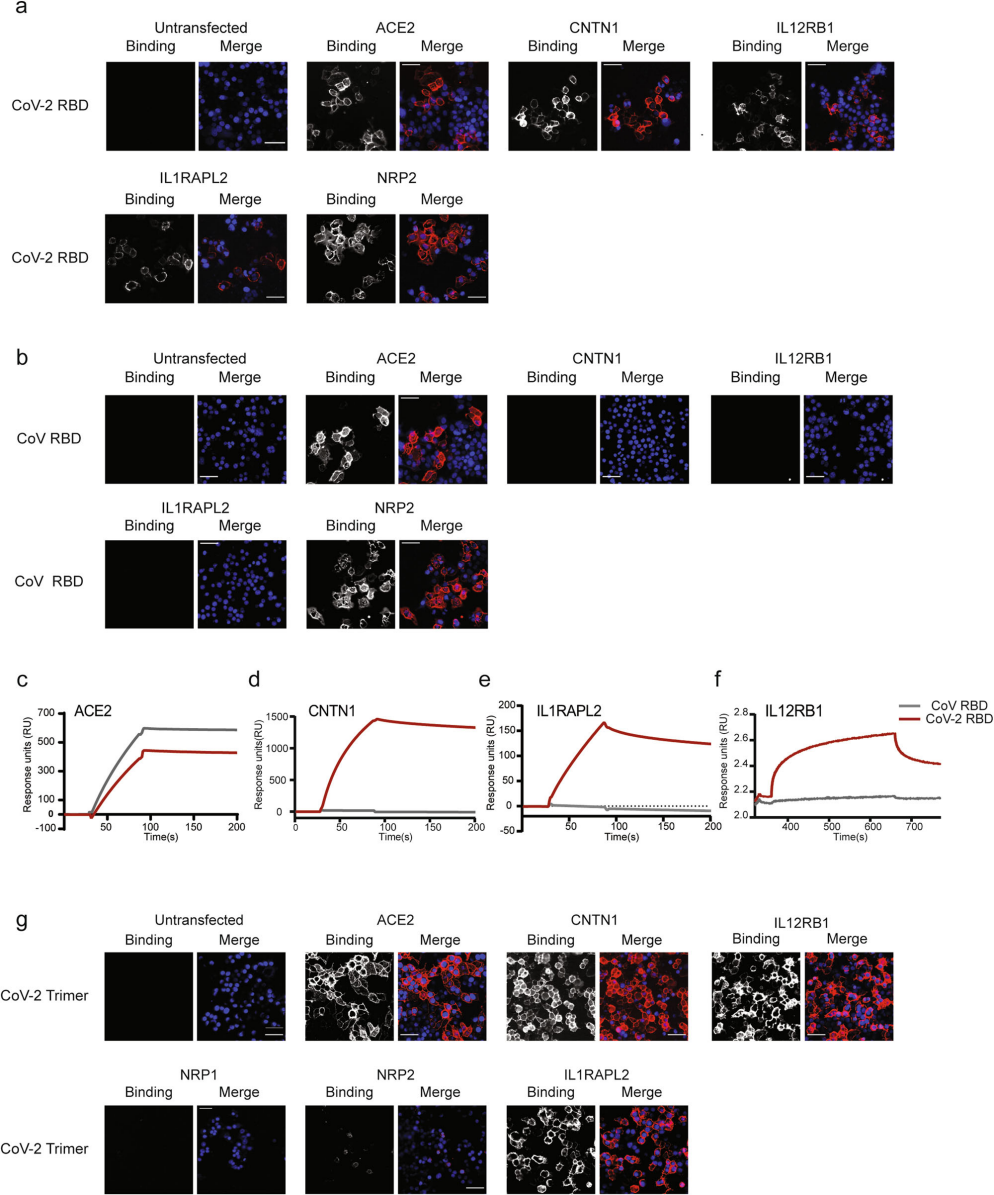
The RBD receptome identifies host cell surface factors specifically targeted by the SARS CoV-2 spike protein. **a** The host proteins identified as high-scoring hits in the screens were expressed on cells as full-length proteins, and binding of the RBD of the SARS CoV-2 spike protein was analyzed by immunofluorescence. **b** The host proteins identified as high-scoring hits in the screens were expressed on cells as full-length proteins, and binding of the RBD of the SARS CoV spike protein was analyzed by immunofluorescence. **a**, **b** RBD tetramer binding to the cell surface at 50 nM concentration is shown. **c**–**f** Analysis of RBD binding to the indicated host factors, as purified proteins, analyzed by surface plasmon resonance or biolayer interferometry. The RBD proteins of SARS CoV-2 (red) or SARS CoV (grey) were immobilized on sensor chips and tested for binding to the indicated binders as recombinant Fc-tagged proteins used as soluble analytes. **f** To test binding to IL12RB1, biotinylated RBD proteins were captured onto streptavidin sensors and analyzed for binding to soluble IL21RB1-Fc protein using biolayer interferometry. The binding of the analytes at 500 nM concentration is shown. **g** Binding of SARS CoV-2 spike trimer to the cellular receptors identified for the RBD. The full ectodomain of the spike trimer was tetramerized and binding to cells expressing the indicated proteins was analyzed by immunofluorescence. Representative images show binding of the spike trimer to the cell surface at 25 nM concentration. Scale bar = 50 μm. In all cases, RBD or trimer binding to cells is shown in red and cell nuclei are depicted in blue.

We then performed a series of validation experiments to confirm these findings using orthogonal approaches. First, the putative RBD binding partners were transiently expressed on cells as either ectodomain-gD-GPI or full-length, native receptors, and the binding of the RBD of the SARS CoV-2 spike to the cell surface of these cells was analyzed by immunofluorescence. Clear binding to the plasma membrane of cells transiently over-expressing native ACE2 was observed, as expected (Fig. 4a). Importantly, these assays also confirmed binding between the RBD of the SARS CoV-2 spike and NRP2, IL12RB1, and CNTN1 when these receptors were expressed as full-length proteins on the plasma membrane (Fig. 4a). These findings were recapitulated when the gD-GPI-tagged receptor ectodomains were expressed on the cells (Supplementary Fig. 2). To further evaluate the specificity of these interactions, similar assays were performed to analyze the binding between the RBD of the SARS CoV spike and the interactors identified for the RBD of the SARS CoV-2 spike. Notably, while these assays confirmed the interaction between ACE2 and NRP2, no detectable binding was observed when the RBD binding partners CNTN1, IL12RB1 and IL1RAPL2 were expressed on the cell surface (Fig. 4b). All receptors were expressed to sufficient levels on the cell surface, as detected by the anti-gD antibody staining on the membrane when the proteins were expressed as gD-GPI-tagged ectodomains (Supplementary Fig. 2). Interestingly, we did not observe detectable binding between either of the SARS CoV RBD proteins and the NRP2-related protein Neuropilin-1 (NRP1), confirming the results obtained in the high throughput screening (Supplementary Fig. 2).

As an additional approach to validate these interactions, surface plasmon resonance analysis was utilized to study binding between the RBD proteins and the cellular receptors identified as binding partners, tested as purified proteins. These assays further confirmed binding and corroborated the specificity of CNTN1, IL12RB1 and IL1RAPL2 host proteins for the RBD of the SARS CoV-2 spike, showing no measurable binding to RBD of the SARS CoV spike in the conditions analyzed (Fig. 4c–f). Finally, we tested if the RBD receptors also interacted with the full ectodomain of the SARS CoV-2 spike trimer, which contained two amino-acid substitutions to eliminate the furin cleavage site (Methods). Similar to the RBD, the spike trimer bound to ACE2, CNTN1, IL12RB1 and IL1RAPL2 expressed on the cell surface (Fig. 4g). We did not observe binding to NRP1, in agreement with published data indicating that binding to NRP1 is mainly mediated by the CendR motif present within the furin cleavage site (mutated in this spike trimer and not present in the RBD protein screened)^17,18^. Interestingly, we observed very weak or negligible binding of the full trimer ectodomain to NRP2, despite this protein interacting with the RBD. This discrepancy indicate that the epitopes involved in the interaction with NRP2 may not be exposed in the full trimer ectodomain tested (stabilized in prefusion conformation), also suggesting differential binding to NRP1 and NRP2 receptors.

### Expression of the host factors targeted by SARS CoV-2 spike

**Fig. 5:**
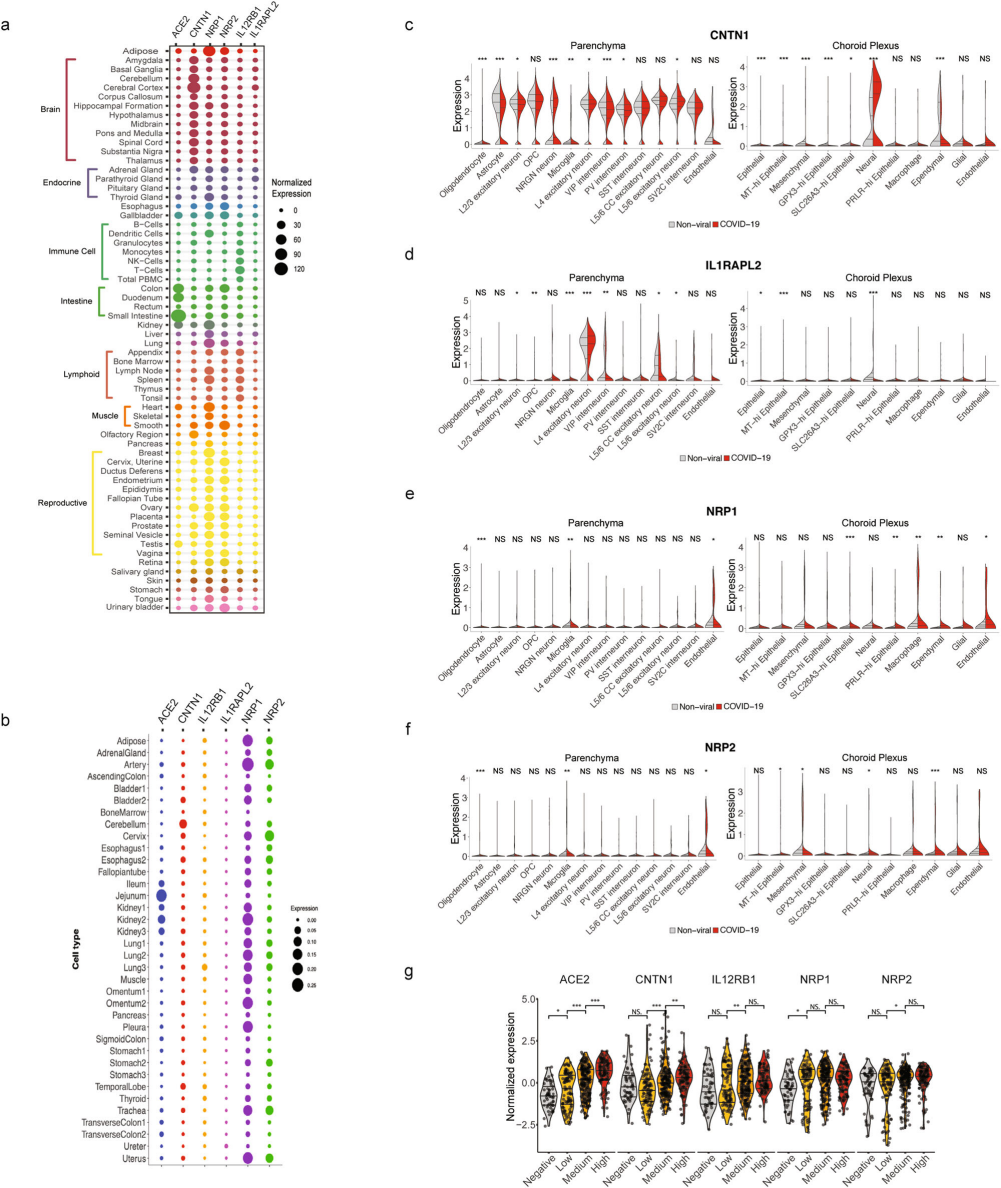
The SARS CoV-2 spike binding partners are expressed in multiple tissues and dysregulated in COVID-19 patients. **a** Expression of SARS CoV-2 spike binding partners across human tissues indicated by different colors (RNAseq data from Human Protein Atlas). **b** Dotplot showing scRNAseq expression of ACE2 and the additional RBD binders IL12RB1, IL1RAPL2, NRP2, CNTN1 and the related protein NRP1 in 36 different tissue types in healthy individuals. scRNAseq data was obtained from GSE13455. **c**–**f** Split violin plots showing the differential gene expression of (**c**) CNTN1; (**d**) IL1RAPL2; (**e**) NRP1 and (**f**) NRP2, between non-viral and COVID-19 infected individuals in each cell type collected from choroid plexus and parenchyma. Statistical significance between low, medium and high viral load is calculated by Mann Whitney U test adjusted by Benjamini-Hochberg correction. *p < 0.05, **p < 0.01, ***p < 0.001. NS, Not significant. Single nucleus RNAseq data for C-F was obtained from twc-stanford.shinyapps.io/scRNA_Brain_COVID19. **g** Violin plots of ACE2, CNTN1, NRP1, NRP2 and IL12RB1 expression by viral load in nasopharynx from healthy individuals and COVID-19 patients (*n* = 430 positive, 54 negative). Each dot represents an individual sample. Statistical significance between low, medium and high viral load is calculated by Mann Whitney U test, *p < 0.05, **p < 0.01, ***p < 0.001. NS, Not significant. RNAseq data was obtained from GSE152075.

Having shown that IL12RB1, IL1RAPL2 and CNTN1 are previously unrecognized cellular factors directly targeted by the SARS-CoV-2 spike, we then analyzed their expression patterns using published databases, both at the tissue level (https://www.proteinatlas.org) as well as in single cell RNA (scRNA) transcriptomes in the major organs. IL1RAPL2 showed overall low expression levels both at the tissue and single cell level in most tissues (Fig. 5a, b), while IL12RB1 was predominantly expressed in immune and lymphoid cells, showing relatively higher expression in scRNA transcriptomes from lung cells (Fig. 5a, b). NRP2 was expressed across multiple tissues, showing relatively higher expression in the digestive and reproductive tract, among others (Fig. 5a, b). Interestingly, CNTN1 was highly and predominantly expressed in nervous system tissue, with similar results at the single cell level (Fig. 5a, b).

A hallmark of the COVID-19 infection is olfactory dysfunction that could be in part explained by the ability of the virus to directly infect the olfactory epithelium^19,20^. To assess whether the newly identified SARS CoV-2 spike binding partners were expressed in this tissue, we queried a recently published scRNA map of the human olfactory tissue^21^. Interestingly, while ACE2 was overall expressed only at low levels, as previously noted, the RBD interactors showed moderate to high expression in this data set. NRP2 was expressed in multiple cell types in the olfactory epithelium, whereas IL12RB1 expression was moderate and limited to the immune cell compartment. In keeping with previous observations, CNTN1 was moderately to highly expressed in different populations of nervous cells, showing the highest representation in stromal cells in this tissue (Supplementary Fig. 3).

### CNTN1 expression is elevated in COVID-19 patients

Next, as a first step to gain insights into the putative roles of the SARS CoV-2 spike binding partners, we investigated the expression of these cellular factors during infection. First, we took advantage of a recent study on single nucleus RNA transcriptomes from brain tissue, including the frontal cortex and choroid plexus, in 14 healthy and 8 COVID-19 patients^22^. As previously noted, and interestingly, ACE2 expression was very low or negligible in these tissues, similarly to IL12RB1 that was virtually absent in these individuals (Supplementary Fig. 4). In turn, IL1RAPL2 and to a lesser extent NRP2 were overall expressed at low levels across cell types in these tissues (Supplementary Fig. 4). By contrast, CNTN1 showed the highest expression levels, particularly in ependymal and neural cells in the choroid plexus and across neuron cell types in the parenchyma (Supplementary Fig. 4). Interestingly, when comparing relative expression patterns in the brain of healthy individuals vs. patients that died from COVID-19, we observed significant differences in the expression of the spike binding partners (Fig. 5c–f), such as upregulation of ILRAPL2 or NRP2 in discrete cell types. More prominently, we observed a marked dysregulation of CNTN1 across cell types in the parenchyma and choroid plexus (Fig. 5c).

Finally, we queried a recently published gene expression dataset representing samples from nasopharyngeal swabs from over 400 COVID-19 patients and healthy controls^23^. In agreement with the low expression levels observed in other tissues, IL1RAPL2 was not detected in these samples. Notably, whereas IL12RB1, NRP1 and NRP2 were only moderately increased as a function of viral load, CNTN1 expression was significantly correlated with viral load in these COVID-19 patients (Fig. 5g and Supplementary Fig. 5), an association that was more prominent in older patients (Supplementary Fig. 5). Interestingly, and similar to CNTN1, ACE2 expression was significantly associated with viral load in this patient cohort (Fig. 5g).

### CNTN1 potentiates ACE2-dependent viral infection

**Fig. 6:**
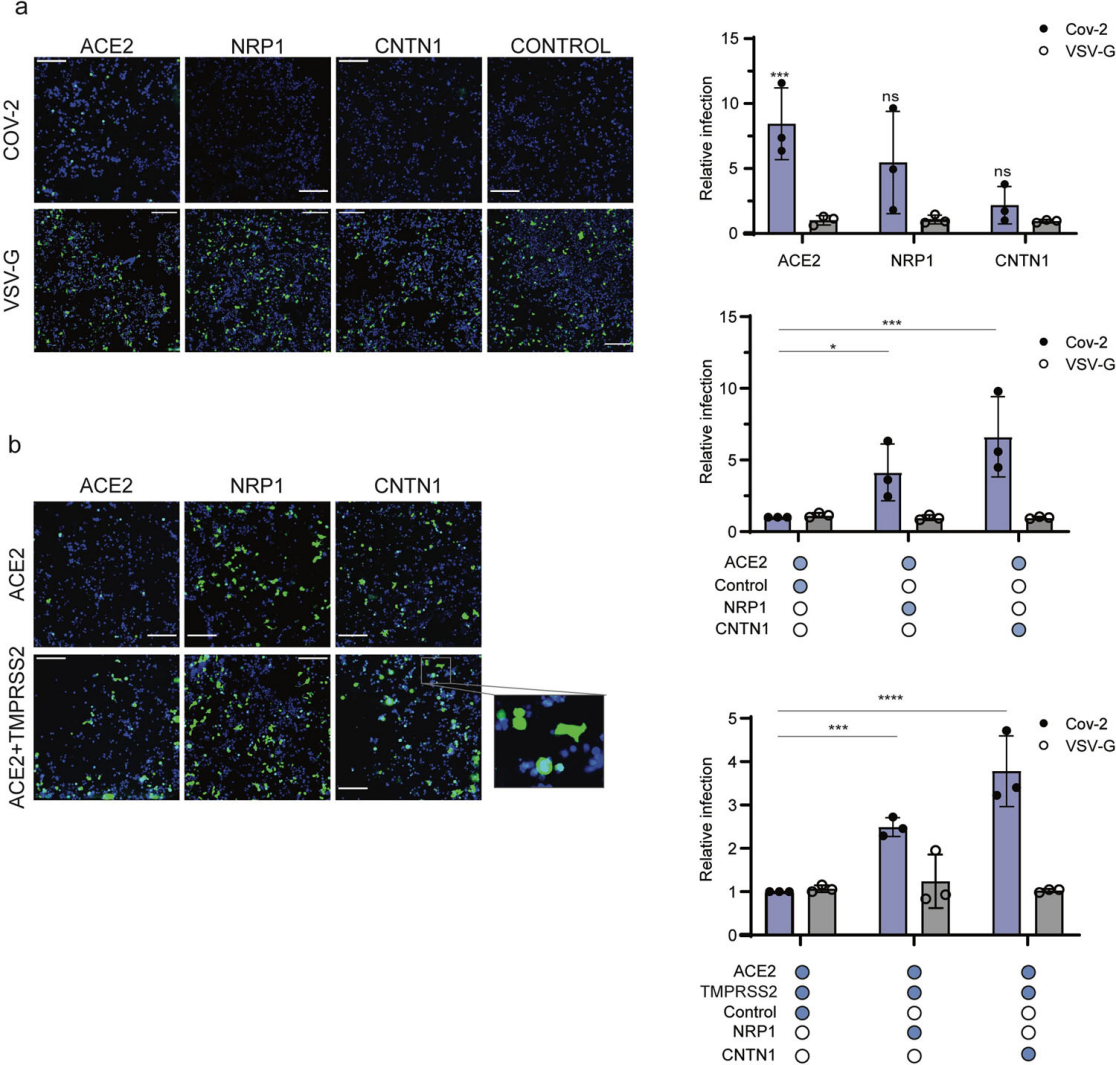
CNTN1 enhances ACE2-dependent entry of SARS-CoV-2 spike pseudotyped particles. **a** Representative images and quantification of SARS CoV-2 pseudotyped particle infection (blue bars) of HEK/293 T cells transiently expressing the RBD receptors ACE2, CNTN1 and NRP1. **b** HEK/293 T cells transiently expressing ACE2, or ACE2 and TMPRSS2 were transfected with CNTN1 or NRP1 and subsequently infected with SARS CoV-2 pseudotyped particles (blue bars). VSV-G pseudotyped infection was used as control (grey bars). Data are normalized to the respective infection of SARS CoV-2 and VSV-G particles in (top graph) control protein-expressing, (middle graph) ACE2-expressing or (bottom graph) ACE2 + TMPRSS2-expressing cells. Infected cells are represented in green; nuclei are depicted in blue. Scale bar = 200 µm. Two-way ANOVA with Sidak’s correction for multiple comparisons; *p < 0.05, *p* < 0.001. NS, not significant. Data shown represents mean ± s.d. from three independent experiments.

Having demonstrated that the SARS CoV-2 spike targets host factors on the cell surface beyond ACE2 and considering that these factors are expressed in relevant tissues during COVID-19 infection, we then investigated their role using spike pseudotyped viral particles as a surrogate for viral entry. To this end, HEK/293 cells, which do not express ACE2, were transfected with plasmids encoding for the newly identified spike binding partners. ACE2 expression made the cells susceptible to infection, as expected, whereas CNTN1 expression by itself promoted low to negligible levels of infection (Fig. 6a). Remarkably, CNTN1 co-expression with ACE2 significantly increased pseudotyped particle infection relative to cells expressing ACE2 alone (Fig. 6b). Furthermore, CNTN1 expression also increased infection in the presence of ACE2 and TMPRSS2, relative to ACE2 and TMPRSS2 alone (Fig. 6b). On the contrary, CNTN1 did not show a significant effect on vesicular stomatitis virus (VSV) glycoprotein-pseudotyped particle infection, used as a control, consistent with the fact that infection by this virus does not rely on ACE2 or CNTN1 for entry (Fig. 6a, b). NRP1, used as a control, did not promote infection but it significantly increased viral entry when co-expressed with ACE2 or ACE2 and TMPRSS2, recapitulating previous observations^17^.

We also evaluated the remaining host factors identified in our SARS CoV spike receptor discovery efforts. Interestingly, the expression of IL12RB1 or IL1RAPL2 did not significantly influence pseudotyped particle entry under the experimental conditions tested (Supplementary Fig. 6), suggesting that these factors may play a role in other aspects of the viral cycle. A hyperinflammatory cytokine storm has been identified as a major cause beneath the severe lung failure and COVID-19 mortality, although the underlying mechanisms of virus-triggered inflammation remain poorly characterized. Modulation of IL-12/IL-23 signaling via the IL12RB1 receptor has been frequently involved in autoimmune disease and bacterial infection^24^, and as such, viral interference with this pathway could play a role in the inflammatory response in the lung, where IL12RB1 is highly expressed. In turn, IL1RAPL2 is a relatively poorly studied cell surface protein that has been associated with functions in the nervous system^25^. In the context of the viral infection, IL1RAPL2 could act as an accessory protein or cofactor for cellular receptors and could influence viral attachment and/or dissemination. Future studies should certainly address the potential functional role of these spike binding partners.

## Discussion

We have developed a cell-based, protein display platform coupled to a tetramer-based screening approach that is optimally suited for in-trans receptor-ligand discovery and that overcomes previous technical hurdles. First, receptor ectodomain tagging enables controlled expression on the cell surface across the human protein library, bypassing the need for often unavailable, and costly, protein-specific antibodies. Our results indicate that a majority of the gD-GPI-tagged receptors are expressed to measurable levels on the cell surface, where they are competent for interaction with the relevant binding partners. Secondly, receptor display in the context of the plasma membrane facilitates native protein conformation and clustering, as well as the addition of post-translational modifications relevant for the function that may be absent in other systems. In addition, tetramerization of the query protein under study allows for improved detection of weak interactions, a main challenge that has limited our understanding of the membrane protein interactomes. This platform is therefore well-suited for high sensitivity and high throughput detection of protein interactions between adjacent membranes, such as those established between the viral envelope and host factors expressed on the cell surface.

The ability of a virus to initiate a productive viral infection and spread in an organism is largely dependent on the recognition of receptors and other factors expressed on the surface of susceptible host cells. This gap is in part due to the scarcity of sensitive technologies focused on the study of the membrane protein interactomes, particularly those that take in between apposed cell membranes. As a crucial step in the viral cycle, the comprehensive characterization of the virus-host interactions that facilitate initial binding and entry into the host cells is essential for the development of new or improved therapeutic options. Although COVID-19 patients primarily manifest with respiratory tract symptoms, a growing number of studies have shown that ACE2 levels are significantly lower in the respiratory tract, relative to other organs such as the kidney, heart or the digestive tract, suggesting that other attachment and/or entry factors expressed in the host cell may compensate low or absence of ACE2 expression. In fact, recent studies have uncovered that NRP1^17,18^ and heparan sulfate^26,27^ modulate SARS CoV-2 entry in an ACE2-dependent fashion, while another report has shown that the tyrosine-protein kinase receptor AXL participates in ACE2-independent entry in a pseudotyped virus infection model^28^. Collectively, these studies support the existence of alternative mechanisms of viral entry that so far remain poorly understood. Together with the continued emergence of SARS-CoV-2 variants harboring mutations in the spike protein, it is paramount to comprehensively characterize the molecular players and mechanisms involved in virus interaction with the host cell.

To the best of our knowledge, our present study provides the first systematic assessment of the host cell surface proteins that are directly targeted by the viral spike, presenting an unbiased evaluation of binding to most single transmembrane-containing proteins in the human genome. This study was performed using a technology optimally suited for the detection of receptor-ligand interactions characterized by a wide range of binding affinities, thus enabling a more exhaustive search for transient interactions that often characterize binding between cellular receptors and proteins expressed on the viral envelope. One of the most remarkable observations in this study is that, in addition to the common receptor ACE2, IL12RB1, ILRAPL2 and CNTN1 are identified as cellular factors that specifically bind to the SARS CoV-2 spike protein, but that do not interact with the SARS CoV counterpart or do so with significantly lower binding affinities. A detailed analysis of the relative binding kinetics may shed light on the role of the spike binding partners and as such it will be of interest to further characterize the biochemistry of these interactions, which has been not addressed in the current study. We did not observe RBD binding to NRP1, previously identified as a viral co-receptor. These data are consistent with the findings that spike binding to NRP1 is mainly mediated by the CendR peptide (682RRAR685) generated upon furin cleavage of the spike^17^. This region is absent in the RBD proteins utilized for our study (spanning R319-S591), thus explaining the lack of binding to NRP1. By contrast, both RBD encoded by SARS CoV and CoV-2 interacted with NRP2 in our assays. However, very weak binding was observed when we tested the full ectodomain of the CoV-2 spike (mutated in the furin cleavage site), possibly indicating that the epitopes that mediate RBD-NRP2 binding are not exposed in the spike trimer. Although out of the scope of this study, these interesting differences suggest that distinct residues are involved in the interaction with NRP1 and NRP2, perhaps implying differential roles during viral infection. Further structural studies are warranted.

Viral proteins can target multiple host factors, often by exploiting multivalent interactions to increase affinity and potency, activities that grant the virus the potential to interfere with several host functions using limited genomic resources^10,29^. It is tempting to speculate that the notable diversity of the extracellular interactome of the SARS-CoV-2 spike might increase the functionality of this protein, influencing the multi-organ tropism of the virus. The neuro-invasive capacity of the virus and its ability to productively replicate in the nervous system remain a matter of debate, and it is unclear whether the neuropathology observed in COVID-19 patients arises from direct virus neuroinvasion, or indirectly, as a consequence of the peripheral infection and associated immune response. Interestingly, we observe that the SARS CoV-2 binding partners are expressed across human tissues, and most prominently, CNTN1 is highly expressed in olfactory tissue and brain, both in bulk tissue samples as well as at the single cell level. Our findings that the nervous cell-associated factor CNTN1 acts as a SARS CoV-2-specific target suggest a possible route by which the virus may interact with cells in the nervous system, which might contribute to the neurological symptoms observed in a high percentage of COVID-19 patients. Our results showing that CNTN1 promotes ACE2-dependent infection echoes previous findings on NRP1, and further supports the existence of additional co-factors that promote viral infection. CNTN1 may play a role in tissues where ACE2 levels are low or negligible, such as certain regions of the brain, where CNTN1 is highly expressed^22,27,30,31^. Alternatively, or in addition, CNTN1 could mediate ACE2-independent routes of infection in vivo in inflamed tissues with high viral load. Although the current study does not address the functional role of this interaction during real SARS CoV-2 infection, the marked increase of CNTN1 in two independent COVID-19 patient cohorts is thought-provoking and strongly suggests a role for this host factor in vivo. Finally, the fact that NRP1 and CNTN1 engage distinct domains (CendR peptide generated upon furin cleavage and full spike ectodomain stabilized in prefusion state, respectively) suggests different mechanisms of action. Emerging evidence have revealed that SARS CoV-2 can engage diverse host factors, possibly through interaction with different domains in the spike, suggesting diverse entry mechanisms in addition to the canonical ACE2-dependent route. It is also tempting to speculate that these factors regulate other aspects of the virus-host interaction beyond entry, such as immune evasion, inflammation or enhanced viral spread.

Although comprehensive, our study is not complete in that it does not address potential interactions with relevant protein families, such as multitransmembrane-containing receptors, transmembrane adhesion molecules or heterodimeric complexes, such as integrins. These proteins have been identified as entry receptors for a number of human viruses, and as such, their potential interactions and functions during SARS CoV infection should be addressed in future studies. Nonetheless, the automated procedure and tetramer-based screening methods developed here should facilitate future studies focused on additional host protein families.

SARS-CoV-2 is the causative agent for the ongoing global pandemic, which has had a devastating socio-economic impact that is also expected to have a long-lasting impact on human health. Understanding basic aspects of viral infection and interaction with the host is paramount to develop new therapeutic options and inform vaccination strategies. As such, future investigations on the role of the SARS CoV-2-specific host targets identified in this study are clearly warranted. More generally, the receptor discovery platform described herein represents an important resource to study virus-host interactions at the molecular level and uncover host targets involved in the recognition of viral pathogens. Ultimately, we anticipate that our findings will empower the development of new or improved therapeutic options against SARS-CoV-2 and other viral threats to global human health.

## Materials and methods

### Cells and transient transfections

HEK/293 T were utilized for the receptor-ligand discovery screens. Cells were grown in DMEM high glucose medium supplemented with 10% FBS, glutamine and antibiotics, and cultured at 37 °C and 5% CO_2_. For transient expression of the screening hits, the cells were transfected in poly-D-lysine-coated 96- or 384-well plates, using Lipofectamine LTX-Plus (Thermo).

### Recombinant proteins

The RBD, PD-L1, PVR, B7-H3 and GDF15 proteins were generated and purified in house. The following proteins were purchased from R&D Systems: IL12RB1-Fc; IL1RAPL2-Fc; CNTN1-Fc. ACE2-Fc was purchased from Sino Biologicals or R&D Systems. B7-H3, PVR are PD-L1 ectodomains, or GDF15 full-length protein, were expressed as Avi-tagged proteins, and purified in-house using standard affinity purification procedures. The proteins were biotinylated using the BirA enzyme following standard experimental procedures described elsewhere^32^. Optimized coding DNAs for SARS-CoV-1 RBD (R319-S591) and SARS-CoV-2 RBD (R319-F541) and spike (M1-Q1208; with substitutions of R682-R685 to GSAS to remove the furin cleavage site and K986-V987 to PP to stabilize the prefusion conformation) were cloned into a pRK vector behind a CMV promoter and, in the case of the RBDs, an N-terminal secretion signal. RBD constructs were generated containing a C-terminal Fc and spike was generated containing a C-terminal trimeric coiled-coil sequence and Avi-His8 tag. DNA constructs were transfected with polyethylenimine using standard protocols into Expi293 cells when the cell density reached 4 × 10^6^ cells per ml and suspension cultures were grown in SMM 293T-I medium under 5% CO_2_ at 37 °C. Culture supernatants were harvested after 6 days, filtered, and subsequently passed over 2 mL of or Protein A or Ni-Excel resin. Resin was washed with ten column volumes of 50 mM Tris pH 8, 100 mM NaCl (with 20 mM imidazole for Ni-Excel) and eluted with the low pH buffer (50 mM Na-citrate pH 3.5) for Protein A or the same buffer containing 250 mM imidazole for Ni-Excel. Samples were adjusted to neutral pH, concentrated and passed over a Superdex 200 16/60 column in 50 mM Tris pH 8, 100 mM NaCl, and peak fractions were pooled. Spike protein trimer was biotinylated using BirA and standard protocols. Following biotinylation of the Avi-tag, spike trimer was subsequently passed over the Superdex 200 16/60 column and peak fractions were pooled and frozen at −80 °C until further use. For the STM interactome discovery screens, the RBD proteins were randomly biotinylated using EZ-Link Sulfo-NHS-Biotin (Cat. No. 21217, Thermo Fisher) following the manufacturer’s protocol with some modifications to minimize biotin incorporation. Following biotinylation, the proteins were tetramerized using APC-conjugated streptavidin (Prozyme) following the protocol described by the NIH tetramer core facility.

### Generation of the ectodomain gD-GPI-tagged protein library

The list of single transmembrane-containing proteins was compiled upon bioinformatics analysis using various algorithms for the prediction of protein features such as protein domains and subcellular localizations, followed by manual curation and review of published annotations, mainly as previously described^5^. The boundaries of the ectodomains were annotated after in silico prediction of the signal peptides and transmembrane regions or GPI-linkage sites. The ectodomain of each receptor, containing its native signal sequence, was synthesized and cloned into a pRK5 vector (Genentech) in frame with a gD-GPI tag. The final library contains 1,188 unique STM receptors, alongside selected receptor isoforms, expressed as ectodomain-gD-GPI fusions. For generation of the full-length clones for cell expression and binding studies, the relevant proteins were cloned into a pRK vector (Genentech) as full-length, untagged, proteins. Full-length and ectodomain-gDGPI plasmids were transiently expressed on HEK/293 T cells, as described.

### Automated single-clone, cell-based, receptor discovery platform

The library of human proteins was expressed on HEK/293 T cells. Cells were transiently transfected with individual receptor clones following a reverse transfection protocol using a semi-automated procedure. Briefly, 25 μL of Lipofectamine LTX-Plus mixture in Opti-MEM medium (Thermo) was dispensed to 384 well microtiter plates containing 6 ng of DNA per well. The DNA-Lipofectamine complexes were incubated for 30 min at 37 °C, and subsequently the cells (resuspended in DMEM media at 0.125 million cells/ml) were aliquoted in the assay plates using an automatic cell dispenser. Screening for RBD binding partners was performed 48 h after transfection. GFP-tagged clones were included to control for cell transfection efficiency.

Analysis of RBD tetramer binding to the cell surface was performed using an integrated robotic system consisting of automated liquid handling devices. Growth media was removed from cell cultures and cells were incubated with the RBD tetramer for 45 min at 4 °C. Cell surface binding was assayed in PBS containing 0.1% BSA supplemented with calcium and magnesium. Following incubation with RBD, the cells were washed and fixed with 4% PFA and stored at 4 °C protected from light. Images were acquired from individual wells using a high-content microscope (In Cell 6000, GE Healthcare). Image data were exported as tiff files and processed using the Developer Toolbox version v1.6 software. Cell surface tetramer staining was represented as fluorescent signal intensities. Images were analyzed using a custom analysis module, and segmentations were performed based on positive cell surface staining. Minimal post-processing analysis and exclusion parameters were set up to obtain optimal outline of desired objects and minimize any background signals due to screening artifacts.

The RBD protein was assayed as an APC-conjugated tetramer to enhance the detection of binding partners due to enhanced avidity. RBD was randomly biotinylated, as described, and subsequently tetramerized following the protocol provided by the NIH Tetramer Core Facility, using fluorescent streptavidin purchased from Prozyme. Streptavidin was added at room temperature with the samples protected from light, and tetramers were subsequently stored on ice until the assay was performed.

### Surface plasmon resonance and Biolayer interferometry

RBD interactions with the relevant host proteins were analyzed by SPR using a Biacore 8 K (GE Healthcare). The RBD proteins were immobilized on CM5 chips using the standard amino coupling method. (GE Healthcare). Analytes were run at the concentrations indicated in each case, in HBS-P buffer (0.01 M HEPES, 0.15 M NaCl and 0.005% surfactant P20, pH7.4). All sensograms were analyzed with BiaEvaluation 4.1 (Biacore) or Proteon Manager 3.1.0.6 (Proteon) software. When indicated, the interactions were analyzed by biolayer interferometry using an Octet-Red system (Sartorius). The biotinylated RBD proteins were loaded onto streptavidin sensors (Sartorius) and tested for binding to recombinant IL12RB1, as soluble analyte in PBS.

### Validation of RBD binding partners by immunofluorescence

The host receptors identified as binding partners for the spike protein were transiently expressed in HEK/293 T cells and assayed after 48 h or 72 h post-transfection. Biotinylated RBD or full-length trimer ectodomain proteins were tetramerized using fluorescence streptavidin (Prozyme) for increased avidity. Typically, the proteins were incubated with the cells for 1 h at 4 °C to avoid internalization, washed and subsequently fixed with 4% PFA. For detection of ectodomain-gD-GPI expression on the cell surface, cells were fixed with 4% PFA, blocked with PBS containing 5% BSA, and subsequently stained using an anti-gD antibody (Abcam). Subsequently, the samples were washed and incubated with fluorescently-labeled alexa fluor antibodies (Thermo Scientific). Incubations with the primary and secondary antibodies were performed in PBS-1% BSA at 4 °C O/N, or 1 h at 37 °C, respectively. Images were acquired using a Leica SP5 confocal or an In Cell 6000 high content imager and analyzed using Fiji software.

### Analysis of tissue expression for RBD binding partners

RNA consensus tissue gene data was downloaded from the Human Protein Atlas webpage (https://www.proteinatlas.org/about/download, HPA v19.3) that contain normalized expression between the HPA, GTEx and FANTOM5 expression datasets. General tissue categories were designated based on GTEx label (https://gtexportal.org) and Tissue Atlas labels.

### Next generation sequencing data analysis

For the single cell RNAseq (scRNAseq) data from healthy individuals, the original digital gene expression (DGE) for different tissue samples reported by Han et al., was obtained from GSE134355 and it was also available in https://figshare.com/articles/HCL_DGE_Data/7235471. Only adult tissues were analyzed. CNTN1 was detected in 36 different tissues. Another human olfactory and respiratory mucosal cell scRNAseq dataset reported by Durante et al.^21^, was collected from GSE139522. The UMAP coordinates and cell cluster annotations were based on the publication. RNAseq dataset of nasopharynx swab from individuals with and without SARS-CoV-2 infection reported by Lieberman et al.^23^, was obtained from GSE152075. The raw count gene expression data was normalized using trimmed mean of M-values (TMM) and transformed with VOOM to log_2_-counts per million with associated precision weights. The association between the normalized, log_2_ transformed gene expression and infection status as well as viral load was analyzed. The infection status and viral load data was obtained from the metadata available in GSE152075. Statistical significance between groups is calculated by Mann Whitney U test. The scRNAseq data of brain and choroid plexus cell types from healthy and COVID-19 infected individuals reported by Yang et al.^22^, was obtained from https://twc-stanford.shinyapps.io/scRNA_Brain_COVID19. The data contained 47,678 droplet-based single-nucleus transcriptomes from the frontal cortex and choroid plexus across 10 non-viral and 4 COVID-19 individuals. 23,626 nuclei across 8 major cell types were profiled in the cortex and 24,052 nuclei across 7 cell types in the choroid plexus. The UMAP coordinates and cell cluster annotations were based on the publication.

### Infection assays with SARS CoV-2 spike pseudotyped particles

Replication incompetent vesicular stomatitis virus (VSV) strain Indiana particles displaying glycoprotein G (VSV-G) and carrying a GFP reporter were purchased from Integral Molecular (Philadelphia, USA). The viral particles pseudotyped with SARS CoV-2 spike protein carrying a GFP reporter were also purchased from Integral Molecular. For the infection assays with the pseudotyped particles, HEK/293 T cells were seeded on M96 or M384 well plates. Cells were transiently transfected with an empty plasmid control or the spike binding partners as full-length, native proteins, and cell cultures were infected 24 h or 48 h post-transfection to enable expression of the cellular receptors. Pseudotyped particles were diluted in serum-free DMEM media and incubated with the cells for 4 h at 37 °C, after which the growth media was replaced with DMEM containing 10% FBS. After 24 h or 48 h postinfection, the cells were washed, fixed with 4% PFA, and stained with DAPI (Thermo Scientific) to visualize nuclei. Images were acquired with a high-content microscope (InCell 6000, GE Healthcare) or Leica SP5 confocal (Leica) at 10x magnification. For quantification of infection, images were analyzed with the InCell Developer software version 4.1. Infection was represented relative to that observed for ACE2- or ACE2 + TMPRSS2-expressing cells in each group. Two-way ANOVA was carried out with Sidak’s correction for multiple comparisons using the GraphPad software v8.

### Reporting summary

Further information on research design is available in the Nature Research Reporting Summary linked to this article.

## Supplementary information

Supplementary Information

Reporting summary

## Data Availability

Data and/or materials are available upon reasonable request, and under a material transfer agreement when applicable.
