# Supplementary material for: Cell-based receptor discovery identifies host factors specifically targeted by the SARS CoV-2 spike
Source: Commun Biol. 2022 Aug 5;5:788. doi: 10.1038/s42003-022-03695-0 (PMC9355963; doi:10.1038/s42003-022-03695-0)
Supplement: Supplementary file 1 — Supplementary Information [file 42003_2022_3695_MOESM1_ESM.pdf]

Supplementary information

Cell-based receptor discovery identifies host factors specifically targeted by the SARS CoV-2 spike

Bushra Husain[1,^], Kobe Yuen[2,^], Dawei Sun[3], Shengya Cao[4], Jian Payandeh[5] and Nadia Martinez-Martin[6,#]

[1]Bayer AG, Portfolio and Assays group, Cologne, Germany.
[2]Genentech, Oncology Biomarker development department, South San Francisco, USA.
[3]Genentech, Structural Biology Department. South San Francisco, USA.
[4]Genentech, Microchemistry, Proteomics and Lipidomics department. Receptor Discovery group. South San Francisco, USA.
[5]Exelixis, South San Francisco, USA.
[6]Regeneron Pharmaceuticals, Infectious Disease department, Tarrytown, New York, USA.

^Authors contributed equally
#Corresponding author
mrtn.nm32@gmail.com

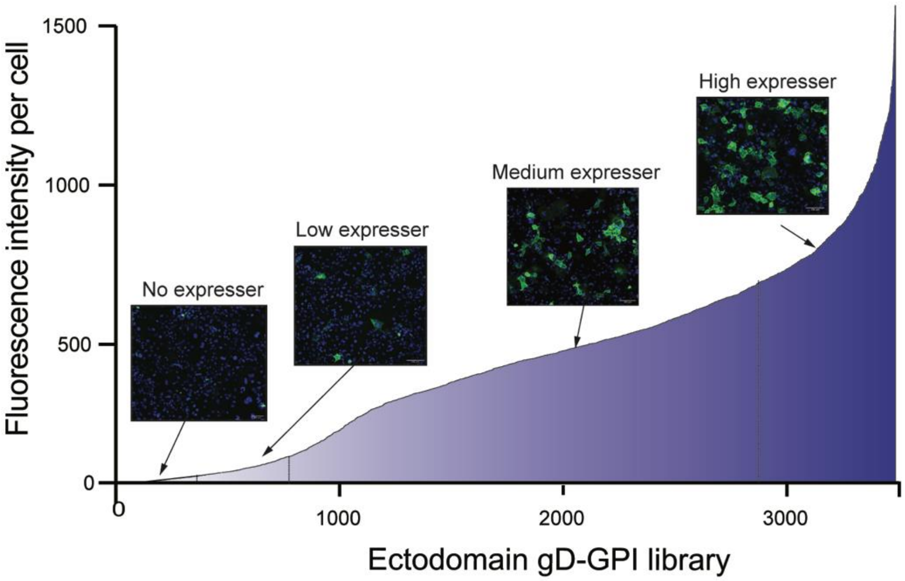

Supplementary Figure 1 (Related to Figure 1 and 2). An automated cell-based platform for enhanced receptor-ligand discovery. Quantification of surface expression of the ectodomain-gD-GPI library using an anti-gD antibody for detection. Representative images of surface staining for not detectable, low, medium, and high expressers are shown. Dotted lines indicate arbitrary cut-offs for the different expression levels. Expression is representative of two independent assays.

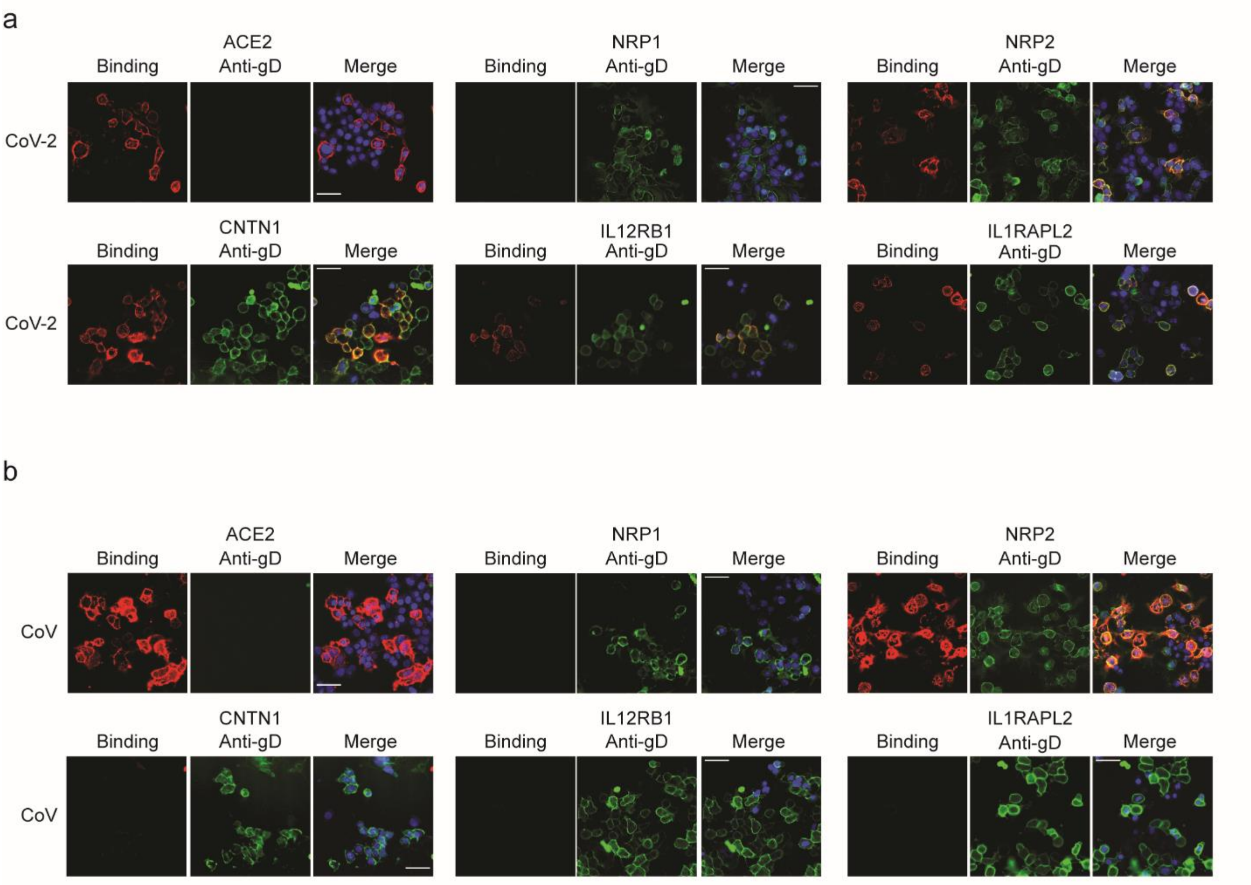

Supplementary Figure 2 (Related to Figure 3 and 4). The RBD receptome identifies host cell surface factors specifically targeted by the SARS CoV-2 spike protein. Binding of the RBD of the **(a)** SARS CoV-2 or **(b)** SARS CoV spike to cells expressing the indicated proteins, expressed as ectodomain-gD-GPI-tagged receptors. ACE2 was expressed as a full-length protein. The RBD proteins were biotinylated and tetramerized, and binding to the cell surface was measured by immunofluorescence. An anti-gD antibody was utilized to analyze expression of the RBD binding partners on the plasma membrane. SARS-CoV RBD did not show detectable binding to NRP1, IL12RB1, IL1RAPL2 or CNTN1 proteins, despite robust expression on the cell surface. Representative images show RBD tetramer binding at 100 nM concentration. RBD binding to the cell is shown in red, nuclei are represented in blue. Expression of the cellular receptors, detected with an anti-gD antibody, is shown in green. Scale bar = 50 µm.

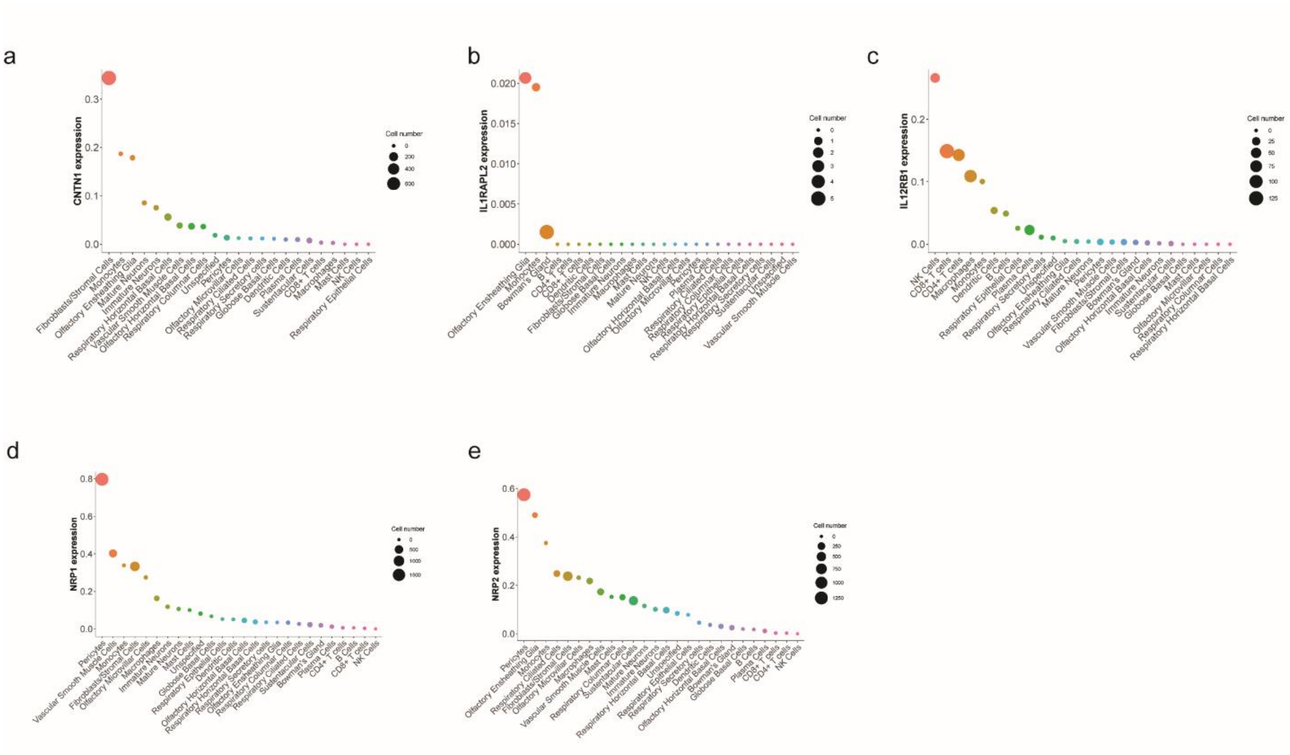

Supplementary Figure 3 (Related to Figure 5). The SARS CoV-2 spike binding partners are expressed in multiple tissues and dysregulated in COVID-19 patients. (**a-e**) Dot plots representing expression of the RBD binding partners in different cell types (n=26) in the olfactory epithelium. Single cell RNAseq data was obtained from GSE139522.

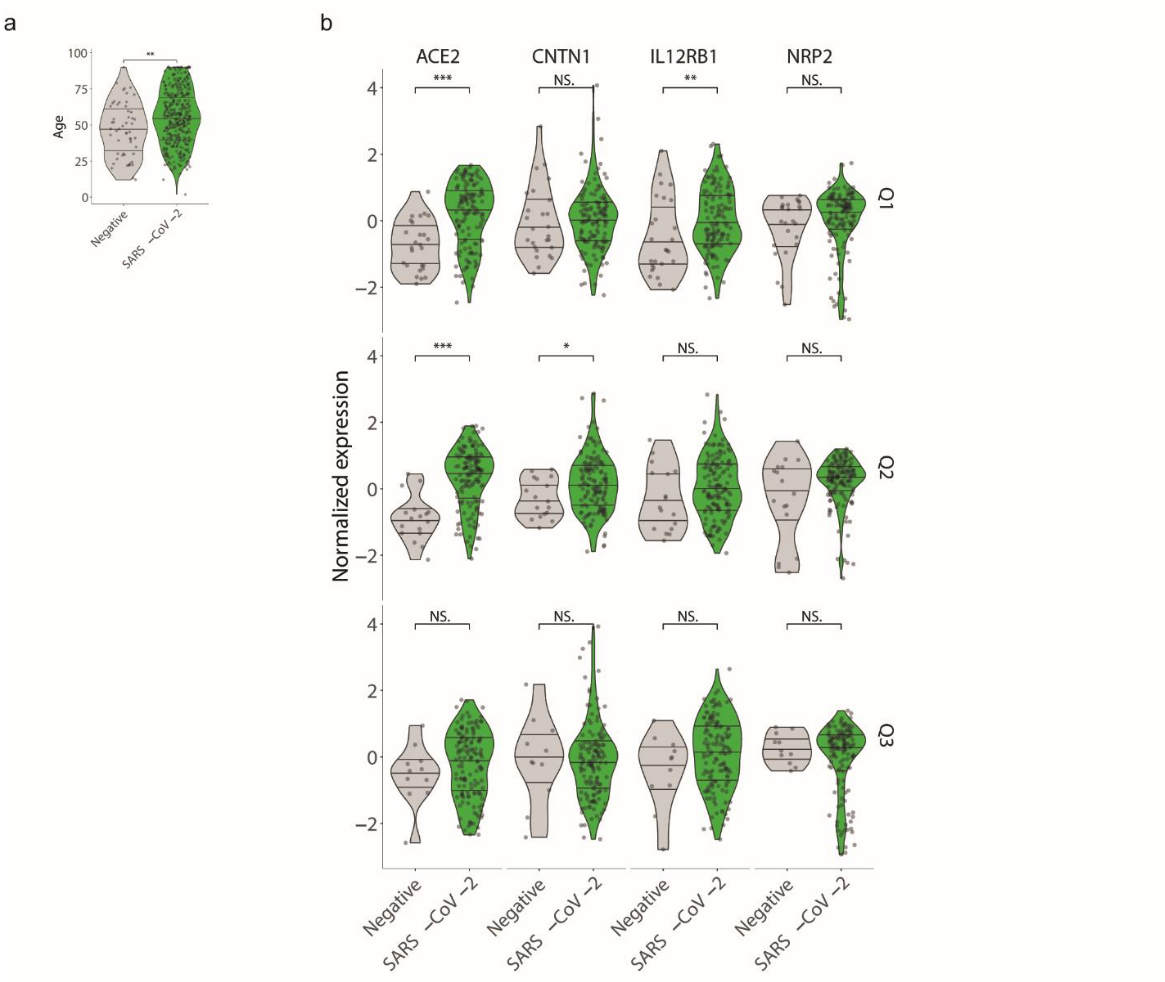

Supplementary Figure 4 (Related to Figure 5). The SARS CoV-2 spike binding partners are expressed in multiple tissues and dysregulated in COVID-19 patients. (**a**) Violin plots showing age of individuals with and without SARS-CoV-2 infection (n = 430 positive, 54 negative). Each dot represents an individual sample. Statistical significance between CoV-2 positive and negative samples is calculated by Mann Whitney U test, **p < 0.01. **(b)** Violin plots comparing the expression levels of ACE2, CNTN1, IL12RB1, IL1RAPL2, NRP1 and NRP2 between SARS-CoV-2 positive and negative samples in stratified by tertiles of ages. Q1: ages 2-45; Q2: ages 46-64; Q3: ages 65-90+. Each dot represents an individual sample. Statistical significance SARS-CoV-2 positive and negative samples is calculated by Mann Whitney U test, *p < 0.05, ***p < 0.001, NS, Not significant. RNAseq data was obtained from GSE152075.

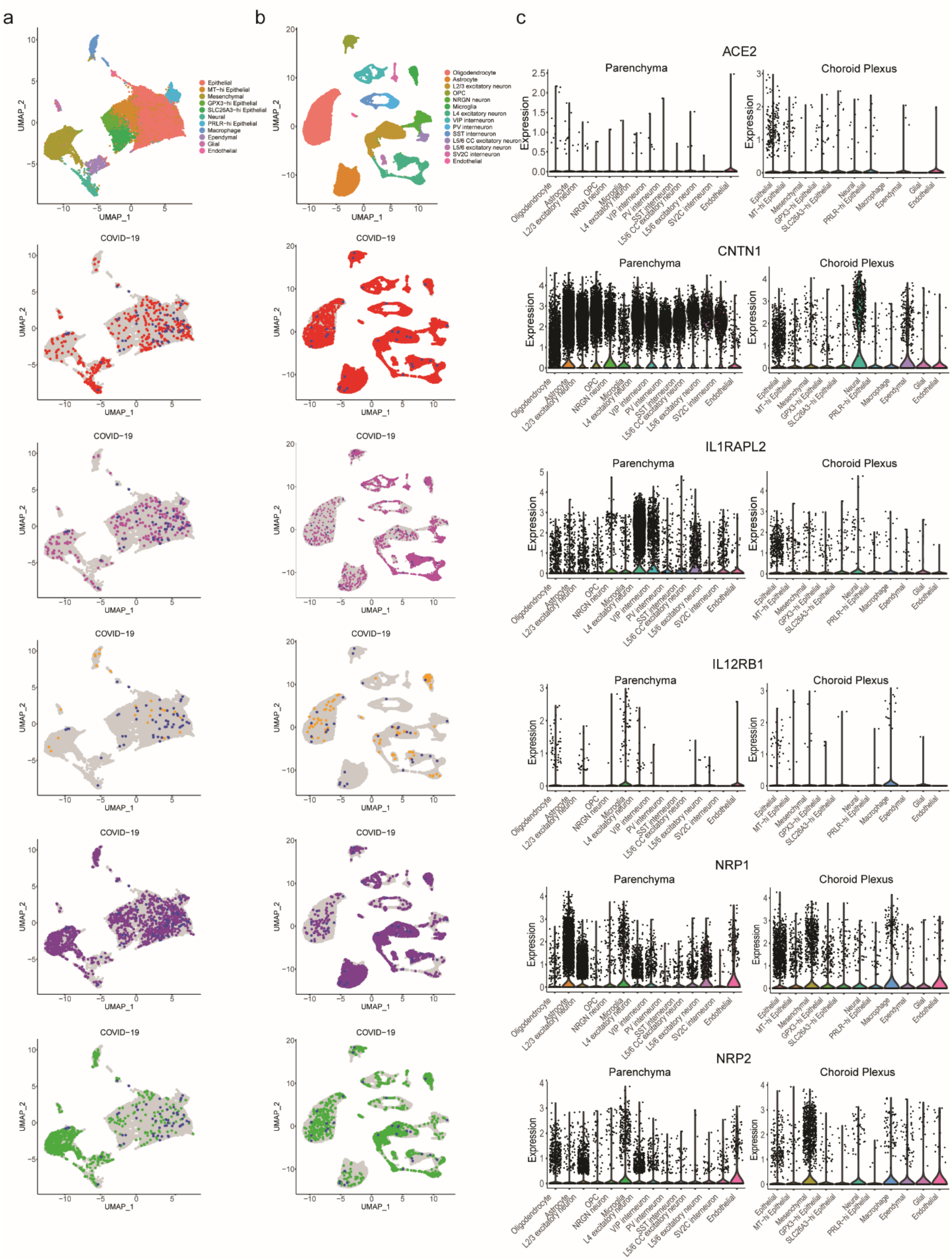

a
b
c
Epithelial
MT−hi Epithelial
Mesenchymal
GPX3−hi Epithelial
SLC26A3−hi Epithelial
Neural
PRLR−hi Epithelial
Macrophage
Ependymal
Glial
Endothelial
Oligodendrocyte
Astrocyte
L2/3 excitatory neuron
OPC
NRGN neuron
Microglia
L4 excitatory neuron
VIP interneuron
PV interneuron
SST interneuron
L5/6 CC excitatory neuron
L5/6 excitatory neuron
SV2C interneuron
Endothelial
UMAP_1
UMAP_2
COVID−19
ACE2
CNTN1
IL1RAPL2
IL12RB1
NRP1
NRP2
Parenchyma
Choroid Plexus
Expression

Supplementary Figure 5 (Related to Figure 5). The SARS CoV-2 spike binding partners are expressed in multiple tissues and dysregulated in COVID-19 patients. (a) Uniform Manifold Approximation and Projection (UMAP) dimensionality reduction plot of 24,072 nuclei across 7 cell types in the choroid plexus. The cell cluster phenotype is noted on the color key legend and labels. UMAPs of ACE2 (blue) and (top to bottom) CNTN1, NRP1, NRP2, IL12RB1 and IL1RAPL2 from choroid plexus. **(b)** UMAP dimensionality reduction plot of 23,626 nuclei across 8 cell types in the cortex parenchyma. The cell cluster phenotype is noted on the color key legend and labels. UMAPs of ACE2 (blue) and (top to bottom) CNTN1, NRP1, NRP2, IL12RB1 and IL1RAPL2 from cortex parenchyma. (**c**) Violin plot showing gene expression of (top to bottom) ACE2, CNTN1, IL1RAPL2, IL12RB1, NRP1 and NRP2 in different cell clusters from choroid plexus and parenchyma tissues. The single nucleus RNAseq data shown were obtained from twc-stanford.shinyapps.io/scRNA_Brain_COVID19.

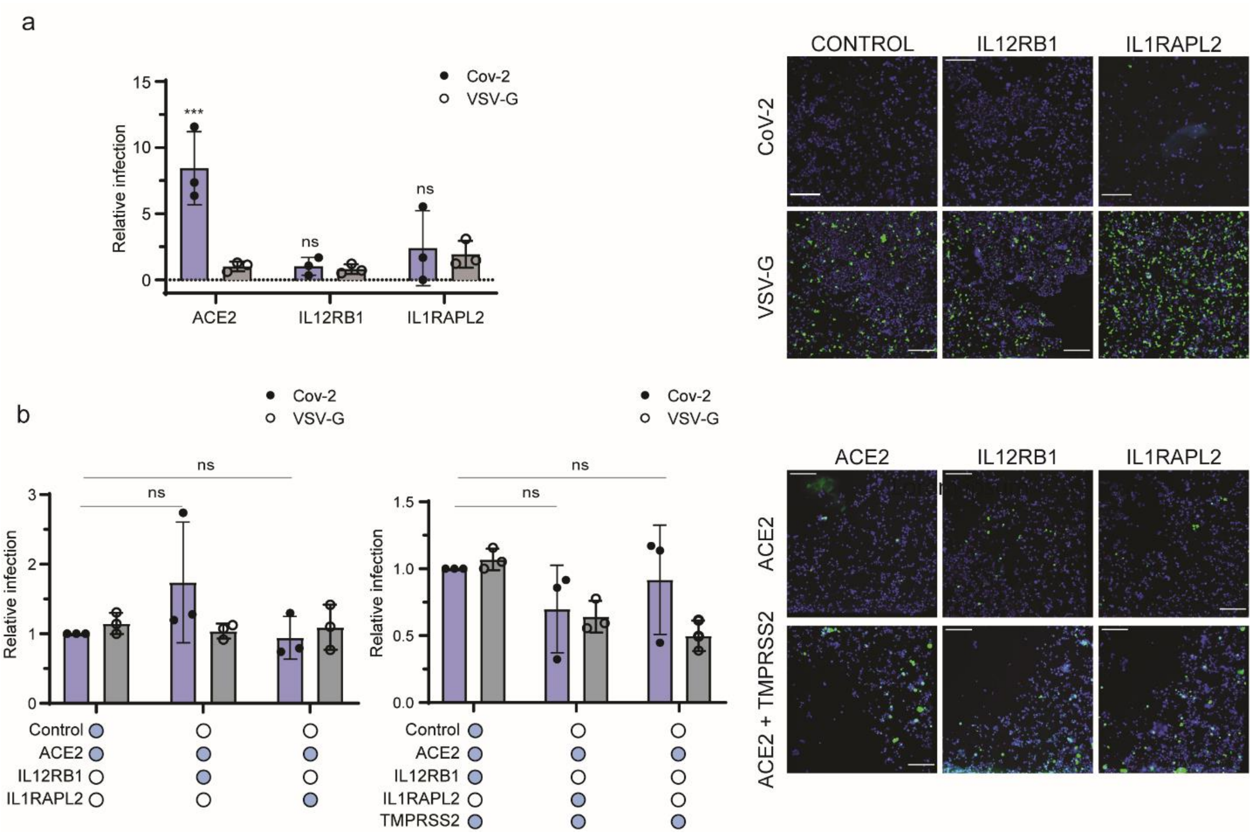

Supplementary Figure 6. Related to Fig. 6. CNTN1 enhances ACE2-dependent entry of SARS-CoV-2 pseudotyped particles. (a) Representative images and quantification of SARS CoV-2 pseudotyped particle infection (blue bars) of HEK/293T cells transiently expressing a control vector or the RBD receptors ACE2, IL12RB1 or IL1RAPL2. VSV-G infectivity was measured as a control (grey bars). Data was normalized to the respective infection of SARS CoV-2 and VSV-G particles in ACE2-expressing cells. (**b**) HEK/293T cells transiently expressing ACE2, or ACE2 and TMPRSS2 were transfected with IL12RB1 or IL1RAPL2, and subsequently infected with SARS CoV-2 pseudotyped particles. No significant differences in infection were observed in the presence of the RBD binders. VSV-G pseudotyped infection was used as control (grey bars). Data are normalized to infection with each of the viruses in (a) control-expressing or (b) ACE2-expressing (left graph) or ACE2+TMPRSS2-expressing (right graph) cells, respectively. Infected cells are represented in green; nuclei are depicted in blue. Scale bar = 200 µm. Two-way ANOVA with Sidak's correction for multiple comparisons; ***$p < 0.001$. NS, not significant. Data shown represents mean ± s.d. from three independent experiments.
