# Supplementary material for: Cell-based receptor discovery identifies host factors specifically targeted by the SARS CoV-2 spike
Source: Commun Biol. 2022 Aug 5;5:788. doi: 10.1038/s42003-022-03695-0 (PMC9355963; doi:10.1038/s42003-022-03695-0)
Supplement: Supplementary file 2 — Reporting summary [file 42003_2022_3695_MOESM2_ESM.pdf]

nature research

Corresponding author(s): Nadia Martinez-Martin

Last updated by author(s): Feb 22, 2022

Reporting Summary

Nature Research wishes to improve the reproducibility of the work that we publish. This form provides structure for consistency and transparency in reporting. For further information on Nature Research policies, see our Editorial Policies and the Editorial Policy Checklist.

Statistics

For all statistical analyses, confirm that the following items are present in the figure legend, table legend, main text, or Methods section.

n/a	Confirmed	
☐	☒	The exact sample size (n) for each experimental group/condition, given as a discrete number and unit of measurement
☐	☒	A statement on whether measurements were taken from distinct samples or whether the same sample was measured repeatedly
☐	☒	The statistical test(s) used AND whether they are one- or two-sided<br>*Only common tests should be described solely by name; describe more complex techniques in the Methods section.*
☒	☐	A description of all covariates tested
☐	☒	A description of any assumptions or corrections, such as tests of normality and adjustment for multiple comparisons
☒	☐	A full description of the statistical parameters including central tendency (e.g. means) or other basic estimates (e.g. regression coefficient) AND variation (e.g. standard deviation) or associated estimates of uncertainty (e.g. confidence intervals)
☐	☒	For null hypothesis testing, the test statistic (e.g. F, t, r) with confidence intervals, effect sizes, degrees of freedom and P value noted<br>*Give P values as exact values whenever suitable.*
☒	☐	For Bayesian analysis, information on the choice of priors and Markov chain Monte Carlo settings
☒	☐	For hierarchical and complex designs, identification of the appropriate level for tests and full reporting of outcomes
☒	☐	Estimates of effect sizes (e.g. Cohen's d, Pearson's r), indicating how they were calculated

Our web collection on statistics for biologists contains articles on many of the points above.

Software and code

Policy information about availability of computer code

Data collection	The data that was used for analysis is publicly accessible in Gene Expression Omnibus (GEO). The GEO accession numbers were available in the corresponding figure legends. Processed single nuclei sequencing data of COVID-19 brain and choroid plexus was obtained from https://twc-stanford.shinyapps.io/scrna_brain_covid19/
Data analysis	No new program was generated. All computational software used for analysis in this manuscript as open source software operated by R Statistical Computing Platform (version 4.0.5)

For manuscripts utilizing custom algorithms or software that are central to the research but not yet described in published literature, software must be made available to editors and reviewers. We strongly encourage code deposition in a community repository (e.g. GitHub). See the Nature Research guidelines for submitting code & software for further information.

Data

Policy information about availability of data

All manuscripts must include a data availability statement. This statement should provide the following information, where applicable:

- Accession codes, unique identifiers, or web links for publicly available datasets
- A list of figures that have associated raw data
- A description of any restrictions on data availability

All Next-Generation Sequencing (NGS) data analyzed in this study is public available on GEO with accession numbers provided in corresponding figure legends. Briefly, single cell RNAseq data of different human tissues was obtained from GSE134355. Single cell RNAseq data of human olfactory tissues was obtained from GSE139522. Bulk RNAseq data of nasopharynx from healthy individuals and covid-19 patients was obtained from GSE152075. Processed single nuclei sequencing data of COVID-19 brain and choroid plexus was obtained from https://twc-stanford.shinyapps.io/scrna_brain_covidl9/. Raw data is available with accession GSE159812.

Field-specific reporting

Please select the one below that is the best fit for your research. If you are not sure, read the appropriate sections before making your selection.

☒ Life sciences ☐ Behavioural & social sciences ☐ Ecological, evolutionary & environmental sciences

For a reference copy of the document with all sections, see nature.com/documents/nr-reporting-summary-flat.pdf

Life sciences study design

All studies must disclose on these points even when the disclosure is negative.

Sample size	Sample size has been indicated in each case. For the bioinformatics analyses, additional information of sample sizes can be found in the respective publications.
Data exclusions	No data was excluded for the bioinformatics or experimental analyses shown in this study.
Replication	The relevant assays were reproduced as indicated in each case.
Randomization	No randomization was performed in the studies reported.
Blinding	Blinding was not possible, data was generated and analyzed in house as part of a Genentech program.

Reporting for specific materials, systems and methods

We require information from authors about some types of materials, experimental systems and methods used in many studies. Here, indicate whether each material, system or method listed is relevant to your study. If you are not sure if a list item applies to your research, read the appropriate section before selecting a response.

Materials & experimental systems

n/a	Involved in the study	
☐	☒	Antibodies
☐	☒	Eukaryotic cell lines
☒	☐	Palaeontology and archaeology
☒	☐	Animals and other organisms
☒	☐	Human research participants
☒	☐	Clinical data
☒	☐	Dual use research of concern

Methods

n/a	Involved in the study	
☒	☐	ChIP-seq
☒	☐	Flow cytometry
☒	☐	MRI-based neuroimaging

Antibodies

Antibodies used	The anti-gD antibody purchased from Abcam, catalog reference ab6507.
Validation	All data related to antibody validation can be found in the supplier's website.

Eukaryotic cell lines

Policy information about cell lines

Cell line source(s)	The cell line HEK293T was obtained from ATCC (ATCC CRL-3216)
Authentication	Standard authentication procedures are performed at ATCC, no further authentication performed.
Mycoplasma contamination	The cell lines were tested for mycoplasma contamination at the Genentech cell culture facility prior to banking, and were confirmed negative.
Commonly misidentified lines (See ICLAC register)	N/A
